# Agitated Psychiatric Patient

**DOI:** 10.21980/J85352

**Published:** 2020-10-15

**Authors:** Brooke M Pabst, Cynthia Leung, Jennifer A Frey, Jennifer Yee

**Affiliations:** * The Ohio State University, Department of Emergency Medicine, Columbus, OH

## Abstract

**Audience:**

This scenario was developed to educate emergency medicine residents about the diagnosis and management of the agitated psychiatric patient.

**Introduction:**

The prevalence of agitation among patients in the emergency department is increasing, with an estimated 1.7 million events occurring annually in the United States.^1^ There are various methodologies for de-escalation, including verbal and chemical de-escalation and physical restraints. Chemical and/or physical restraints are sometimes necessary to ensure patient and staff safety when verbal de-escalation is ineffective, particularly since agitation is the leading cause of hospital staff injuries.^2^ Chemical restraints have been shown to be less physically traumatizing to patients.^3^ ^4^ Adverse events associated with physical restraints include persistent psychological distress, blunt chest trauma, aspiration, respiratory depression, and asphyxiation leading to cardiac arrest.^5^ In regards to chemical restraints, adverse event reporting has been heterogeneous among studies, but the most consistent reported events involve respiratory compromise such as desaturation, airway obstruction, and respiratory depression.^3^ A study measuring QTc (corrected QT interval) after high-dose intramuscular ziprasidone or haloperidol did not demonstrate any QTc longer than 480 msec.^6^ Other events linked to chemical restraints include uncommon cardiovascular events and extrapyramidal side effects from medications.^3^ The main classes of medications utilized for chemical restraint include first-generation antipsychotics (eg, haloperidol and droperidol), second-generation antipsychotics (olanzapine, quetiapine, risperidone, aripiprazole, and ziprasidone), benzodiazipenes (eg, lorazepam and midazolam), and N-methyl-D-aspartic acid (NMDA) receptor antagonists (eg, ketamine).^7^,^8^ It is important to exclude other medical causes of agitation, consider the differential diagnoses, and then select a medication that is tailored to address underlying etiologies while remaining cognizant of the side effect profiles of these chemical agents.

**Educational Objectives**: At the conclusion of the simulation session, learners will be able to: 1) Obtain a relevant focused history and physical examination on the agitated psychiatric patient. 2) Develop a differential for the agitated psychiatric patient, including primary psychiatric conditions and other organic pathologies. 3) Discuss the management of the agitated psychiatric patient, including the different options available for chemical sedation. 4) Prioritize safety of self and staff when caring for an agitated psychiatric patient.

**Educational Methods:**

This session was conducted using simulation with a standardized patient, followed by a debriefing session and lecture on the presentation, differential diagnosis, and management of the agitated psychiatric patient. Debriefing methods may be left to the discretion of participants, but the authors have utilized advocacy-inquiry techniques. This scenario may also be run as an oral board examination case.

**Research Methods:**

The residents are provided a survey at the completion of the debriefing session to rate different aspects of the simulation, as well as provide qualitative feedback on the scenario. This survey is specific to the local institution’s simulation center.

**Results:**

Feedback from the residents was overwhelmingly positive, although many stated that they felt some degree of intimidation or stress from the standardized patient who did not break from their role throughout the scenario.

The local institution’s simulation center feedback form is based on the Center of Medical Simulation’s Debriefing Assessment for Simulation in Healthcare (DASH) Student Version Short Form^9^ with the inclusion of required qualitative feedback if an element was scored less than a 6 or 7. This session received mostly 7 scores (extremely effective/outstanding).

**Discussion:**

This is a physically safe method for reviewing management of the agitated psychiatric patient. There are multiple potential presentations of the agitated psychiatric patient, as well as varying underlying etiologies. These scenarios may be tailored to the needs of the learner, including identifying agitation, pharmacologic review, and de-escalation techniques.

**Topics:**

Medical simulation, agitated psychiatric patient, chemical sedation, verbal de-escalation, emergency medicine, psychiatry.

## USER GUIDE

**Table:** 

List of Resources:	
Abstract	59
User Guide	61
Instructor Materials	65
Operator Materials	71
Debriefing and Evaluation Pearls	75
Simulation Assessment	79

**Learner Audience:**

Medical students, interns, junior residents, senior residents

**Time Required for Implementation:**

Instructor Preparation: 30 minutes

Time for case: 20 minutes

Time for debriefing: 40 minutes

**Recommended Number of Learners per Instructor:**

3–4

**Topics:**

Medical simulation, agitated psychiatric patient, chemical sedation, verbal de-escalation, emergency medicine, psychiatry.

**Objectives:**

By the end of this simulation session, the learner will be able to:

1. Obtain a relevant focused history and physical examination on the agitated psychiatric patient.
2. Develop a differential for the agitated psychiatric patient, including primary psychiatric conditions and other organic pathologies.
3. Discuss the management of the agitated psychiatric patient including the available options for chemical sedation.
4. Prioritize safety of self and staff when caring for an agitated psychiatric patient.

### Linked objectives and methods

Psychiatric patients with agitation require prompt recognition, treatment, and evaluation by a psychiatrist once medically cleared of suspected acute underlying somatic conditions. In this case, providers will review how to quickly assess and diagnose the agitated psychiatric patient with an appropriately focused history and physical exam (*objective 1*) while still considering other differentials that may present similarly (*objective 2*). Participants will administer appropriate chemical sedation when verbal de-escalation techniques are ineffective (*objective 3*) in order to prioritize the safety of self and staff, as well as the patient (*objective 4*).

This simulation scenario allows learners to reinforce their agitated psychiatric patient management skills in a psychologically safe learning environment, and then receive formative feedback on their performance.

### Recommended pre-reading for instructor

The authors recommend instructors review literature regarding the agitated psychiatric patient, including epidemiology, presenting signs/symptoms, diagnosis, and management. Suggested readings include materials listed below under the “References/suggestions for further reading” section. References 1–3 and 8 are suggested as “top reads” by the authors.

### Results and tips for successful implementation

This simulation was written to be performed as a simulation scenario, but it also may be used as a mock oral board case. The case was written for emergency medicine residents. A similar agitated psychiatric simulation case was conducted for approximately 35 emergency medicine residents during November and December 2019. The majority of the residents found the simulation case to be an engaging context for learning, provoking in discussion of management, and had appropriate reflections for how their assessment and management could improve. Debriefing topics included the differential diagnosis of agitation, the clinical presentation of the agitated psychiatric patient, different chemical sedation agents used for agitation, discussion of verbal de-escalation techniques, use of isolation rooms if available, the importance of patient reassessment, and prioritizing the safety of patient and staff members.

The local institution’s simulation center feedback form is based on the Center of Medical Simulation’s Debriefing Assessment for Simulation in Healthcare (DASH) Student Version Short Form^9^ with the inclusion of required qualitative feedback if an element was scored less than a 6 or 7:

1. Before the simulation, the instructor set the stage for an engaging learning experience. The instructor introduced him/herself, described the simulation environment, what would be expected during the activity, and introduced the learning objectives: mean was 6.33.
2. During the simulation, the instructor maintained an engaging context for learning. The instructor clarified the purpose of the debriefing, what was expected of me, and the instructor’s role in the debriefing. The instructor acknowledged concerns about realism and helped me learn even though the case(s) were simulated: mean was 6.38.
3. The instructor structured the debriefing in an organized way. The conversation progressed logically rather than jumping around from point to point. Near the beginning of the debriefing, I was encouraged to share my genuine reactions to the case(s) and the instructor seemed to take my remarks seriously. In the middle, the instructor helped me analyze actions and thought processes as we reviewed the case(s). At the end of the debriefing, there was a summary phase where the instructor helped tie observations together and relate the case(s) to ways I can improve my future clinical practice: mean was 6.52.
4. The instructor provoked in-depth discussions that led me to reflect on my performance. The instructor used concrete examples--not just abstract or generalized comments--to get me to think about my performance. The instructor’s point of view was clear; I didn’t have to guess what the instructor was thinking. The instructor listened and made people feel heard by trying to include everyone, paraphrasing, and using non-verbal actions like eye contact and nodding, etc: mean was 6.52.
5. The instructor identified what I did well or poorly - and why. I received concrete feedback on my performance or that of the team based on the instructor’s honest and accurate view. The instructor helped explore what I was thinking or trying to accomplish at key moments: mean was 6.43.
6. The instructor helped me see how to improve or how to sustain good performance: mean was 6.43.

We utilized a standardized patient from our institution’s standardized patient program, which are paid positions. We had the standardized patients playing security, nursing, and the patient come in for thirty minutes a week prior to running the scenario to practice their scripts and ensure responses were appropriate. Teaching goals for the learners were reviewed with the standardized patients so they knew what our expectations were for the scenario. For a more cost-effective method, volunteer confederates may be used, but the authors strongly encourage faculty to rehearse the scenario ahead of time with whomever is participating. Scripts used are included at the end of this section.

A majority of residents described the simulation scenario as “intense,” since we encouraged the standardized patient (SP) to be intimidating. The SP would stand very close to the residents, occasionally yelling or swearing, and would throw soft objects around the room but away from any of the participants. We encouraged the SP to also improvise, including walking up to a resident who had a stethoscope around their neck and threatening to strangle them. Depending on the facilitator’s audience, this may not be a scenario appropriate to junior learners (such as medical students) who are not familiar with simulation but may be of benefit for senior medical students interested in emergency medicine or psychiatry.

Residents voiced that they deviated from their typical clinical practice in this simulation scenario because they would typically stop attempts at verbal de-escalation earlier and leave the room sooner if they felt unsafe, but they did not know if this would disrupt the scenario. Running this case as an in-situ scenario may decrease some of these artificial behaviors.

Many residents struggled with identifying alternatives to haloperidol or lorazepam, which is what the local institution often uses for agitated patients. This led to a productive discussion on atypical antipsychotics, management of agitation related to alcohol intoxication versus withdrawal, and treatment of agitated delirium. Some teams did not voice a suspicion for a hidden weapon on the patient when the SP was dressed in street clothes, and therefore discussion then focused on the importance of having the patient change into a gown for staff and patient safety. We also reviewed how items in the room or located on staff may be used as a weapon, including neckties, lanyards, and stethoscopes. Teams discussed how their management might change if they had fewer resources available, such as at a freestanding emergency department (ED) without security staff present, and facilitators may choose to modify this case to reflect such an environment.

### Agitated Psychiatric Patient Script

Initially sitting, then start standing and yelling louder.

Start standing in front of residents, clenching fists intermittently with feet apart, intermittently pacing.

Occasionally attempt to walk towards door.

Take (unbreakable) objects and throw them onto the floor away from any people (such as a pillow).

Initially answer in short sentences (yes/no, if possible) while sitting.

If asked to be placed on the cardiac monitor or have a peripheral intravenous catheter (IV) placed, say, “I already told you, you’re not putting anything (in/on) me.”

As residents ask about your psychiatric history, begin to stand, clench your fists, and devolve into general anger/agitation (in this order).

- “Man, I don’t need to be here.”
- “I’m not crazy-let me out of this place!”
- “The police have it out for me. I was just minding my own business”
- “I’m not doing any tests” “I’m gonna call a lawyer and sue you. You are violating my rights!”
- “you’ll be sorry. You’ll see.”
- “You’re messing with the wrong guy, just wait.”
- “You’re not sticking me with any needles.”
- (once security is mentioned) – “I will punch him in the face!”
- (after security helps to verbally de-escalate) – “Fine. I’ll take the shot if then you all just leave me alone.”

### Security Scripts

- “Hello, I’m (Roger).”
- (as needed) – “Hey, would you be able to take a few steps back? We want to make sure everyone stays safe.”
- “The doctors just want to make sure you’re doing all right.”
- “I’m not here to hurt you, just to make sure that everyone stays safe.”

## Figures and Tables

**Table:** 

**Vitals:**	Heart Rate (HR) 120	Blood Pressure (BP) 190/100	Respiratory Rate (RR) 25
	Temperature (T) 98.0°F	Oxygen Saturation (O_2_Sat) 98%	

**Table:** SIMULATION EVENTS TABLE:

Minute (state)	Participant action/trigger	Patient status (simulator response) & operator prompts	Monitor display (vital signs)
0:00 (Baseline)	Patient moved into trauma bay in the emergency department by police.	Learners should begin by reviewing initial vitals and introducing themselves to the patient.	T 98.0 °F HR 120 BP 190/100 RR 18 O2 98% RA
4:00	Patient should not be on a monitor. Nurses were able to get an initial set of vitals, but unable to attach telemetry leads. Still obtaining history from patient.	Patient initially answers in short sentences (yes/no, if possible). Then devolve into general anger/agitation. (in this order) - “Man, I don’t need to be here.” - “I’m not crazy- let me out of this place!” - “I’m not doing any tests!” - “I’m gonna call a lawyer and sue you. You are violating my rights!” - **“**You’ll be sorry. You’ll see” - “You’re messing with the wrong guy, just wait.” - (once security is mentioned) – “I will punch him in the face!”	T 98.0 °F HR 120 BP 190/100 RR 18 O2 98% RA
8:00	Attempt to obtain focused physical examination.	Initially the patient begins the encounter by sitting, then they will stand and begin yelling louder. He will stand in front of learners, clenching fists intermittently with feet apart, intermittently pacing, and holding his hand over his pocket to signify he may have a hidden weapon. The patient will occasionally attempt to walk towards door and take (unbreakable) objects and throw them onto the floor away from any people (such as a pillow).	T 98.0 °F HR 120 BP 190/100 RR 18 O2 98% RA
12:00	Call for security and initiate chemical sedation.	Have security come within 1 minute of being called. Security will talk the patient into cooperating and changing into a gown. Nurse will ask if learners still want chemical sedation medications 1) Resident should ask for appropriate IM chemical sedation. 2) If they ask nursing to give medications before security is there, have the nurse state they don’t feel comfortable with his level of agitation. 3) If security is not called, have the patient start specifically threatening the learners verbally: “If you don’t let me go, I don’t know what is going to happen.” “Let me go before things get really ugly.”	T 98.0 °F HR 120 BP 190/100 RR 18 O2 98% RA

**Table:** 

**Critical Actions:** Review triage vitals, including temperature Attempt initial verbal de-escalation Prioritize changing the patient into a gown Ask for security to be present prior to administering IM medications Order chemical sedation medication Demonstrate awareness of exit strategies	0:00

**Table:** 

	Milestone	Did not achieve level 1	Level 1	Level 2	Level 3
1	**Emergency Stabilization (PC1)**	□ Did not achieve Level 1	□ Recognizes abnormal vital signs	□ Recognizes an unstable patient, requiring intervention Performs primary assessment Discerns data to formulate a diagnostic impression/plan	□ Manages and prioritizes critical actions in a critically ill patient Reassesses after implementing a stabilizing intervention
2	**Performance of focused history and physical (PC2)**	□ Did not achieve Level 1	□ Performs a reliable, comprehensive history and physical exam	□ Performs and communicates a focused history and physical exam based on chief complaint and urgent issues	□ Prioritizes essential components of history and physical exam given dynamic circumstances
3	**Diagnostic studies (PC3)**	□ Did not achieve Level 1	□ Determines the necessity of diagnostic studies	□ Orders appropriate diagnostic studies. Performs appropriate bedside diagnostic studies/procedures	□ Prioritizes essential testing Interprets results of diagnostic studies Reviews risks, benefits, contraindications, and alternatives to a diagnostic study or procedure
4	**Diagnosis (PC4)**	□ Did not achieve Level 1	□ Considers a list of potential diagnoses	□ Considers an appropriate list of potential diagnosis May or may not make correct diagnosis	□ Makes the appropriate diagnosis Considers other potential diagnoses, avoiding premature closure
5	**Pharmacotherapy (PC5)**	□ Did not achieve Level 1	□ Asks patient for drug allergies	□ Selects an medication for therapeutic intervention, consider potential adverse effects	□ Selects the most appropriate medication and understands mechanism of action, effect, and potential side effects Considers and recognizes drug-drug interactions
6	**Observation and reassessment (PC6)**	□ Did not achieve Level 1	□ Reevaluates patient at least one time during case	□ Reevaluates patient after most therapeutic interventions	□ Consistently evaluates the effectiveness of therapies at appropriate intervals
7	**Disposition (PC7)**	□ Did not achieve Level 1	□ Appropriately selects whether to admit or discharge the patient	□ Appropriately selects whether to admit or discharge Involves the expertise of some of the appropriate specialists	□ Educates the patient appropriately about their disposition Assigns patient to an appropriate level of care (ICU/Tele/Floor) Involves expertise of all appropriate specialists
9	**General Approach to Procedures (PC9)**	□ Did not achieve Level 1	□ Identifies pertinent anatomy and physiology for a procedure Uses appropriate Universal Precautions	□ Obtains informed consent Knows indications, contraindications, anatomic landmarks, equipment, anesthetic and procedural technique, and potential complications for common ED procedures	□ Determines a back-up strategy if initial attempts are unsuccessful Correctly interprets results of diagnostic procedure
20	**Professional Values (PROF1)**	□ Did not achieve Level 1	□ Demonstrates caring, honest behavior	□ Exhibits compassion, respect, sensitivity and responsiveness	□ Develops alternative care plans when patients’ personal beliefs and decisions preclude standard care
22	**Patient centered communication (ICS1)**	□ Did not achieve level 1	□ Establishes rapport and demonstrates empathy to patient (and family) Listens effectively	□ Elicits patient’s reason for seeking health care	□ Manages patient expectations in a manner that minimizes potential for stress, conflict, and misunderstanding. Effectively communicates with vulnerable populations, (at risk patients and families)
23	**Team management (ICS2)**	□ Did not achieve level 1	□ Recognizes other members of the patient care team during case (nurse, techs)	□ Communicates pertinent information to other healthcare colleagues	□ Communicates a clear, succinct, and appropriate handoff with specialists and other colleagues Communicates effectively with ancillary staff

## INSTRUCTOR MATERIALS

**Case Title:** Agitated Psychiatric Patient

**Case Description & Diagnosis (short synopsis):** Patient is Reggie Stone, who is a 25-year-old male who presents by police for erratic behavior. He has a past medical history of bipolar disorder, schizophrenia, and anxiety. He was standing in the middle of the street, waving a large knife at passersby and yelling about topics with religious overtones. Police took his knife away prior to arrival to the hospital. Patient allowed triage staff to obtain an initial set of vitals, but not to place an IV or obtain blood work. He will initially begrudgingly answer questions, but he will not get into a gown if asked. After a few minutes, he will not answer questions. He will become more agitated, standing over residents and swearing loudly. Ideal management is to call security, order chemical sedation, and keep staff and themselves safe. Non-ideal management is to try verbal de-escalation without pharmacological agents for sedation, insist that the nurses give intramuscular (IM) medications without security nearby to physically restrain the patient as needed, and/or try to restrain the patient themselves. If these situations occur, the patient will begin holding his hand over his pocket as if to suggest he has a concealed weapon, as well as throwing soft objects around the room. Participants should also evaluate for underlying organic conditions such as serotonin syndrome, neuroleptic malignant syndrome, delirium, and concurrent overdose. Anything ordered will be pending and not obtained, including point-of-care glucose. Case will end after patient is appropriately sedated with IM medications with security present or at facilitator discretion.

### Equipment or Props Needed

- At least three standardized patients or confederates for the following roles: a nurse, 1–2 security members, as well as the agitated patient
- A piece of paper with initial vitals printed on them
- Simulated medications in vials or syringes with labeling: haloperidol, diphenhydramine, ziprasidone, lorazepam, ketamine
- Folded gown and pillow to be available to throw onto the floor

### Confederates needed

Primary nurse, 1–2 security guards, patient

### Stimulus Inventory

**Background and brief information:** Patient is Reggie Stone, who is a 25-year-old male who presents by police for erratic behavior.

**Initial presentation:** Patient is Reggie Stone, a 25-year-old male, who presents by police for erratic behavior. He was standing in the middle of the street, waving a large knife at passersby and yelling about topics with religious overtones. Police took his knife away prior to arrival to the hospital.

- Past medical history: bipolar disorder, schizophrenia, and anxiety
- Past surgical history: none
- Medications: unknown, lithium in the past
- Allergies: none
- Family history: noncontributory
- Vital signs:
  ○ HR – 120 beats per minute
  ○ Resp rate – 20 breaths per minute
  ○ Temp – 98.0 °F
  ○ BP – 190/100 mmHg
  ○ Pulse ox – 98% on room air
- Weight: 220 lbs./100 kg

Assessment: Patient is dressed in street clothes. He is verbally agitated and sitting in a chair.

**How the scene unfolds:** Patient is Reggie Stone, a 25-year-old male who presents by police for erratic behavior. He has a past medical history of bipolar disorder, schizophrenia, and anxiety. He was standing in the middle of the street, waving a large knife at passersby and yelling about topics with religious overtones. Police took his knife away prior to arrival to the hospital. If nursing is asked, they may report that the chart has revealed that he has been admitted here in the past for psychosis and has a history of suicidal attempts, specifically via lithium overdose. Patient allowed triage to obtain an initial set of vitals, but not to place an IV or obtain blood work. He will initially begrudgingly answer questions but will not get into a gown if asked. He will initially answer in short sentences, particularly giving yes/no answers. This will then evolve into anger and agitation saying, “Man, I don’t need to be here,” “I’m not crazy – let me out of this place!” “I’m not doing any tests!” “I’m gonna call a lawyer and sue you. You are violating my rights!” **“**You’ll be sorry. You’ll see.” and “You’re messing with the wrong guy, just wait.” Once security is mentioned, the patient will say “I will punch him in the face!”

They will be unable to get the patient onto the monitor. Nurses were able to get an initial set of vitals and a past medical history from the patient but were not able to attach cardiac monitoring leads due to the patient’s agitation. Vitals should be printed on paper to demonstrate static initial readings from triage. On assessment, patient is alert and tachycardic. He is agitated and psychotic. He will initially tolerate a brief physical exam, but he will become irritated with a prolonged neurologic exam. Patient begins the case initially sitting. The patient will start standing and yelling louder when he is requested to get into a gown or to have blood work drawn. He will then stand in front of residents, clenching fists intermittently with feet apart, intermittently pacing. Occasionally he will attempt to walk towards door. He will take unbreakable objects (such as a pillow or plastic chairs) and throw them onto the floor away from any people.

After a few minutes, he will refuse to answer any additional questions. He will become more agitated, standing over residents and yelling/swearing. Ideal management is to call security, order chemical sedation medications, and keep staff and themselves safe. Security guards enter the room within 1 minute of being called. The nurse will then ask if residents still want sedation medications. Learners will then be able to convince the patient into changing into a gown after sedatives have been administered.

Ineffective management by the residents includes attempting verbal de-escalation without pharmacological agents for sedation, insisting the nurse gives IM medications without security nearby to physically restrain the patient as needed, and/or trying to restrain the patient themselves. If they ask nursing to administer IM medications, nursing will state they are uncomfortable doing so unless security is present. If any of these aforementioned situations occur, the patient will begin holding his hand over his pocket as if to suggest he has a concealed weapon, as well as throwing soft objects around the room. Participants should also evaluate for underlying organic conditions such as serotonin syndrome, neuroleptic malignant syndrome, delirium, and concurrent overdose. Anything ordered will be pending and not obtained, including point-of-care glucose. Case will end after patient is appropriately sedated with IM medications with security present or at facilitator discretion.

### Critical actions

1. Review triage vitals, including temperature
2. Attempt initial verbal de-escalation
3. Prioritize changing the patient into a gown
4. Ask for security to be present prior to administering IM medications
5. Order chemical sedation medications
6. Demonstrate awareness of exit strategies

**Case Title:** Agitated Psychiatric Patient

**Chief Complaint:** Patient is Reggie Stone, a 25-year-old male, who presents by police for erratic behavior.

**General Appearance:** Alert, verbally agitated, sitting in a chair.

### Primary Survey

- **Airway:** intact
- **Breathing:** normal, clear bilaterally
- **Circulation:** tachycardic rate and regular rhythm. 2+ symmetric radial pulses bilaterally

### History

- **History of present illness:** Patient is Reggie Stone, who is a 25-year-old male, who presents by police for erratic behavior. If asked, he has a past medical history of bipolar disorder, schizophrenia, and anxiety. He was standing in the middle of the street, waving a large knife at passersby and yelling about topics with religious overtones. If asked, police did take his knife away prior to arrival to the hospital. If asked, he has been admitted here in the past for psychosis and has a history of suicide attempts, specifically overdosed on lithium in the past.
- **Past medical history**: bipolar disorder, schizophrenia, and anxiety
- **Past surgical history:** none
- **Medications:** unknown, lithium in the past
- **Allergies:** none
- **Social history:** occasional alcohol use, smokes tobacco, denies illicit drug use other than occasional marijuana use
- **Family history:** noncontributory

### Primary Assessment

- **Vital signs**:
  ○ HR – 120 beats per minute
  ○ Resp rate – 20 breaths per minute
  ○ Temp – 98.0 °F
  ○ BP – 190/100 mmHg
  ○ Pulse ox – 98% on room air
- **Weight:** 220 lbs./100 kg
- **Assessment:** Patient is alert. He is verbally agitated and sitting in a chair.

### Secondary Survey/Physical Examination

- **General appearance:** Alert, agitated, sitting in a chair dressed in street clothes
- **HEENT:**
  ○ **Head:** within normal limits
  ○ **Eyes:** within normal limits. No ocular clonus, normal pupils, pupils are 3mm, bilaterally symmetric.
  ○ **Ears:** within normal limits
  ○ **Nose:** within normal limits
  ○ **Throat:** within normal limits
- **Neck:** within normal limits
- **Heart:** regular and tachycardic, otherwise within normal limits
- **Lungs:** within normal limits
- **Abdominal/GI:** within normal limits. No abdominal distention or tenderness. Normal bowel sounds present.
- **Genitourinary:** deferred
- **Rectal:** deferred
- **Extremities:** within normal limits
- **Back:** within normal limits
- **Neuro:** within normal limits. Ambulating around the room without difficulty. Refuses detailed neurologic exam (sensory, clonus, reflexes).
- **Skin:** within normal limits. No diaphoresis
- **Lymph:** within normal limits
- **Psych:** verbal agitation, intense eye contact, limited insight and judgment. denies suicidal ideation.

## OPERATOR MATERIALS

### Diagnosis

Agitated Psychiatric Patient

### Disposition

Admission to Inpatient Psychiatric Unit

### Agitated Psychiatric Patient Script

Initially sitting, then start standing and yelling louder.

Start standing in front of residents, clenching fists intermittently with feet apart, intermittently pacing.

Occasionally attempt to walk towards door.

Take (unbreakable) objects and throw them onto the floor away from any people (such as a pillow).

Initially answer in short sentences (yes/no, if possible) while sitting.

If asked to be placed on the cardiac monitor or have a peripheral intravenous catheter (IV) placed, say, “I already told you, you’re not putting anything (in/on) me.”

As residents ask about your psychiatric history, begin to stand, clench your fists, and devolve into general anger/agitation (in this order).

- “Man, I don’t need to be here.”
- “I’m not crazy- let me out of this place!”
- “The police have it out for me. I was just minding my own business”
- “I’m not doing any tests” “I’m gonna call a lawyer and sue you. You are violating my rights!”
- “you’ll be sorry. You’ll see.”
- “You’re messing with the wrong guy, just wait.”
- “You’re not sticking me with any needles.”
- (once security is mentioned) – “I will punch him in the face!”
- (after security helps to verbally de-escalate) – “Fine. I’ll take the shot if then you all just leave me alone.”

### Security Scripts

- “Hello, I’m (Roger).”
- (as needed) – “Hey, would you be able to take a few steps back? We want to make sure everyone stays safe.”
- “The doctors just want to make sure you’re doing all right.”
- “I’m not here to hurt you, just to make sure that everyone stays safe.”

## DEBRIEFING AND EVALUATION PEARLS

### Agitated Psychiatric Patient

- The agitated psychiatric patient is frequently seen in the Emergency Department and the incidence is increasing.
- It is important to rapidly obtain a relevant focused history and physical examination on the agitated psychiatric patient.
- De-escalation techniques include non-pharmacological approaches as well as chemical sedation and should be employed on a case-by-case basis.
  ▪ Non-pharmacological approaches include verbal de-escalation, quieting the room, and dimming the lights.
- Physical restraints are linked to more adverse events such as lasting psychological distress, blunt chest trauma, aspiration, respiratory depression, and asphyxiation leading to cardiac arrest.^5^
- There are four main classes of chemical sedation agents^7^,^8^ and it is important to tailor the choice of medication to the patient’s presentation, underlying medical conditions and pharmacokinetic profile since there is no single “best” agent or combination for every situation.
  ▪ First-generation antipsychotics (eg, haloperidol and droperidol)
  ▪ Second-generation antipsychotics (olanzapine, quetiapine, risperidone, aripiprazole, and ziprasidone)
  ▪ Benzodiazepines (eg, lorazepam and midazolam)
  ▪ NMDA receptor antagonists (eg, ketamine)
- When choosing a pharmacologic agent, it is important to consider the underlying cause of the patient’s agitation, any other medical conditions or home medications that may affect pharmacokinetics, the efficacy of the medication, and to weigh the risks and benefits of each medication class.
  ▪ In a systematic review, the efficacy among these medications used for chemical sedation supported better outcomes of combination therapy with antipsychotics and benzodiazepines compared to a single agent alone. 3
    - Combination therapy of antipsychotic plus benzodiazepine required fewer repeated doses, produced fewer adverse events, and sedated more patients within 15–20 minutes compared to benzodiazepines alone.^3^
    - Benzodiazepine monotherapy was linked to over- or under-sedation, increased need for redosing, and respiratory compromise.^3^
- ▪ Intramuscular (IM) ketamine has a rapid onset of action (approximately 3 minutes) and a favorable safety profile. It does not depress respiratory drive or depress the gag reflex at typical dosing.^10^
  - IM Ketamine can be used as a single agent with faster onset of sedation compared to the traditional combination of haloperidol and lorazepam.^5^,^11^
  - An important side effect of Ketamine is laryngospasm.^12^
  - Mean duration of action of IM ketamine is 5–30 minutes.^12^
- ▪ Among antipsychotics, IM olanzapine is favorable since its success rate as a single agent within 1 hour of use is 80–85% and when used in combination therapy, it requires lower dosing of benzodiazepine when combined with midazolam with no significant increase in adverse events.^13^
- There are several toxidromes and diagnoses to consider for the agitated psychiatric patient, and a differential diagnosis list may aid in the appropriate selection of chemical sedation.
  ▪ It is important to identify delirium in the acutely agitated patient since it suggests a potentially reversible underlying etiology to be addressed.
    Address medical causes such as head trauma, hypoglycemia, or medication reactions.
  ▪ Benzodiazepines are helpful in cases of neuroleptic malignant syndrome^14^,^15^, serotonin syndrome^16^, alcohol withdrawal^17^,^18^, anticholinergic toxicity^17^, and excited delirium.^18^
    Benzodiazepines are contraindicated in patients with significantly diminished level of consciousness or alcohol intoxication due to the additive effect of respiratory depression.
      In patients with contraindications to benzodiazepines, you can consider using dexmedetomidine or ketamine^10^,^13^,^18^,^29^ as alternative agents, or an antipsychotic medication in the case of alcohol intoxication with acute agitation.^8^
    ▪ In cases of delirium that is not due to alcohol, benzodiazepine withdrawal, or sleep deprivation, second-generation antipsychotics are the preferred agents for chemical sedation.^8^
    ▪ Antipsychotics are useful in treating primary psychosis or psychiatric illnesses.^8^
  Along with the differential of agitation, it is important to consider the favorable or unfavorable aspects of your chemical agent.
    ▪ Among first-generation antipsychotics, both haloperidol and droperidol have minimal effects on vital signs, negligible anticholinergic activity, and minimal interactions with nonpsychiatric mediations.^8^
  First-generation antipsychotics have important neurologic side effects including extrapyramidal side effects (EPS) such as dystonia, akathisia, and catatonia, as well as neuroleptic malignant syndrome.
    EPS may be reduced with the addition of lorazepam, diphenhydramine, or promethazine.^8^
    The newer second-generation antipsychotics such as olanzapine and quetiapine have much lower rates of EPS.^8^
  Droperidol, an older agent, is not currently an FDA approved medication for agitated psychiatric patients due to a black box warning for QTc prolongation.
    - Data regarding the consequences of this prolongation have been controversial and it is still in clinical use in anesthesia practice.
    - Of note, other first-generation antipsychotics such as haloperidol and various psychiatric medications can prolong the QTc. Using these medications, especially with repeated use and in combination with other medications that may prolong the QTc, should be used cautiously with recommendation for serial ECGs.
    - However, one study showed that high-dose IM doses of ziprasidone and haloperidol did not show any QTc prolongation greater than 450msec.^6^ The need to administer antipsychotics for patient/staff safety should be prioritized with considerations of the patient’s current medication regimen; however, there is no universal recommendation to delay chemical sedation in an acutely agitated patient in order to first obtain an ECG.
    - More recent data have supported the use of droperidol in the emergency department, since it has not only been as efficacious but also safe to treat patients with acute psychosis and remains an option with shared decision making.^21^
  ▪ Barbiturates and opiates are less optimal agents because they have higher adverse effects such as depressed mental status and apnea.^3^
- Prioritizing staff and patient safety requires rapid assessment and treatment of the agitated patient.
- Involve security, if available, for use of chemical and/or physical restraints. Ensure patient’s belongings and clothing are removed in order to unmask potential weapons and to prevent harmful use of clothing or accessories, such as strangulation.
- Do not allow an agitated patient to get between you and an exit strategy.
- Always position yourself with a clear and un-obstructed exit, such as a door. If there is a possibility that a patient is or may become agitated, you may evaluate them from the doorway – ideally with security nearby. It is a good idea to be cognizant of all physical exits and potential physical barriers before entering a room.

### Other debriefing points

Closed-loop communication amongst team: Was it used? Why or why not? Were there any implications of this during case execution?

## SIMULATION ASSESSMENT

*Agitated Psychiatric Patient*

Learner: _________________________________________

***Assessment Timeline***

This timeline is to help observers assess their learners. It allows observer to make notes on when learners performed various tasks, which can help guide debriefing discussion.

**Critical Actions:**

□ Review triage vitals, including temperature
□ Attempt initial verbal de-escalation
□ Prioritize changing the patient into a gown
□ Ask for security to be present prior to administering IM medications
□ Order chemical sedation medication
□ Demonstrate awareness of exit strategies

**Summative and formative comments:**

**Milestones assessment:**
